# Nanoparticle-Based Devices in the Control of Antibiotic Resistant Bacteria

**DOI:** 10.3389/fmicb.2020.563821

**Published:** 2020-11-25

**Authors:** Mario F. Gómez-Núñez, Mariel Castillo-López, Fernando Sevilla-Castillo, Oscar J. Roque-Reyes, Fernanda Romero-Lechuga, Diana I. Medina-Santos, Ricardo Martínez-Daniel, Alberto N. Peón

**Affiliations:** ^1^Laboratory of Biomedicine Santiago Ramón y Cajal, Sociedad Española de Beneficencia, Pachuca, Mexico; ^2^Área Académica de Medicina, Universidad Autónoma del Estado de Hidalgo, Pachuca, Mexico; ^3^Facultad de Medicina, Universidad Nacional Autónoma de México, Mexico, Mexico; ^4^Laboratorio de Microbiología, Escuela Superior de Apan, Universidad Autónoma del Estado de Hidalgo, Pachuca, Mexico

**Keywords:** gold nanoparticles, silver nanoparticles, graphene nanoparticles, nanoparticle toxicity, nanoparticle modified devices, antibiotic resistant bacteria

## Abstract

Antimicrobial resistance (AR) is one of the most important public health challenges worldwide as it represents a serious complication that is able to increase the mortality, morbidity, disability, hospital stay and economic burden related to infectious diseases. As such, the spread of AR–pathogens must be considered as an emergency, and interdisciplinary approaches must be undertaken in order to develop not only drugs, but holistic strategies to undermine the epidemic and pathogenic potentials of multi-drug resistant (MDR) pathogens. One of such approaches has focused on the use of antimicrobial nanoparticles (ANPs), as they have demonstrated to possess strong antimicrobial effects on MDR pathogens. On the other hand, the ability of bacteria to develop resistance to such agents is minimal. In this way, ANPs may seem a good choice for the development of new drugs, but there is no certainty about their safety, which may delay its translation to the clinical setting. As MDR pathogens are quickly becoming more prevalent and drug development is slow and expensive, there is an increasing need for the rapid development of new strategies to control such agents. We hereby explore the possibility of designing ANP-based devices such as surgical masks and fabrics, wound dressings, catheters, prostheses, dentifrices, water filters, and nanoparticle-coated metals to exploit the potential of such materials in the combat of MDR pathogens, with a good potential for translation into the clinical setting.

## Introduction

Antibiotics have been considered among the most important discoveries in the medical field as they are of great importance to combat infectious diseases, significantly reducing the total morbidity and mortality of such conditions. However, the misuse, and even the use, of such compounds creates antibiotic resistance (AR), which is a bacterium’s ability to keep living and/or reproducing in high antibiotic concentrations where other bacteria of the same species become inactive or die. Bacteria can be intrinsically resistant to certain antibiotics but can also develop resistance to antibiotics via genetic mutations. Furthermore, they can also communicate resistance genes (r genes) by horizontal gene transfer (HGT) to other bacteria, which enhances the spreading potential of this phenomenon (Munita and Arias, 2016). Moreover, bacteria can become resistant to multiple drugs at the same time, being termed multi-drug resistant (MDR) bacteria, or informally “super bugs.” Any bacteria can become MDR, but there is a group that has an enhanced ability to develop this condition, and is comprised by *Enterococcus faecium, Staphylococcus aureus, Klebsiella pneumoniae, Acinetobacter baumannii, Pseudomonas aeruginosa*, and *Enterobacter* spp., which are collectively known as the “ESKAPE” group of pathogens (Rice, 2008).

Antibiotic resistance-pathogens are a serious problem because they enhance mortality and morbidity rates, increase the risks of medical procedures and medical costs per procedure, prolong illness and convalescence periods, and attack preferentially immunocompromised and hospitalized patients, complicating their conditions (Tanwar et al., 2014), overall summing to a great economic burden worldwide. On the other hand, AR also represents a serious impairment on modern medicine’s ability to treat and prevent bacterial infections. As a consequence, AR is currently considered one of the principal threats to global public health by the World Health Organization (WHO; World Health Organization [WHO],, 2018) and the Centers of Disease Control (CDC; Center of Disease Control,, 2020), and both Organizations have strongly recommended the development of new strategies to cope with the problem.

The community has responded by implementing regulations on antimicrobial use and availability, active surveillance on AR-strains and antibiotic use, educational programs for both the medical practitioners and the general public, improved sanitation, and even banning antibiotic use for growth promotion in animals and improving farm biosecurity. Nonetheless, such political and social measures are yet to produce a full control on the phenomenon. As a consequence, new antimicrobial agents and derivatives, anti-virulence drugs, ecologic and evolutionary management approaches, and even new therapeutic options like those derived from bacteriophages, enzybiotics, and antimicrobial nanoparticles (ANPs) have been undertaken (Roca et al., 2015). Some of these strategies are, apparently, good candidates for the control of the whole phenomenon, but nanoparticles (NPs) are of special interest as they have shown little potential for the development of bacterial resistance against them; despite this, they have not been shown to be entirely safe for their use as drugs. Nonetheless, they may represent a valuable tool to exert ecological control of AR-bacteria, because of their enhanced residual activity. As such, in this review we will examine the antimicrobial properties of NPs and we will discuss their potential to design devices that may be useful in the control of AR-pathogens when used, not as drugs, but as barriers in the clinical, veterinary, farm and sewage settings.

## Where is the Root of the Problem?

As early as three years after penicillin was introduced into the market and its use was widespread, penicillin-resistant bacteria became a reality and rendered a new challenge for drug developers, whom responded by developing new antibacterial agents. Such increased rate of antibiotic discovery led the period of 1950–1970 to be considered as the “golden age” of antibiotic discovery. Nonetheless, resistance to antibiotics developed shortly after the introduction of every new class of antimicrobial compound (Davies and Davies, 2010; Zaman et al., 2017), regardless of the mode of action of each antibiotic. Although new interesting approaches to antibiotic development have been successfully applied in the experimental setting (Ling et al., 2015; Stokes et al., 2020), worldwide experience with such drugs foresees the development of resistance against these new options, as resistance to any antibiotic class to date has been detected, and even multiple mechanisms of resistance against each type of compound have been described.

In the beginning of the aforementioned “antibiotic era” AR was an uncommon finding, but such phenomenon has spread slowly and steadily all over the world. In 2019 more than 2.8 million AR-infections were detected only in the United States (US), and such infections produced more than 35,000 deaths (Solomon and Oliver, 2014), while in Europe more than 33,000 people die yearly due to AR-infections, and it is thought that the phenomenon will lead to ten million extra human deaths worldwide by 2050 (Abat et al., 2017). Moreover, AR is not an exclusive phenomenon of developed countries as 43% of the deaths due to nosocomial infections in Thailand occur by MDR-pathogens (Lim et al., 2016). The economic burden of this type of infections is also important as only in the US over 20 billion dollars are lost per year, and worldwide economic losses of more than $100 trillion on a yearly basis have also been reported (Munita and Arias, 2016). In such panorama WHO’s (World Health Organization [WHO],, 2018) and CDC’s (Center of Disease Control,, 2020) words of caution on the matter are more than justified.

Despite these evidences, the conception of AR as a pandemic is debatable. For instance, a true epidemic represents a rapid increase in the number of cases, and a pandemic is just a global epidemic. As such, pandemics and epidemics represent a sudden impact on the economy and public health, but they do not impact on the long term any of the aforementioned phenomena. On the opposite end, an endemic may occur in high numbers, but its rate is constant or changes slowly and steadily, and may represent a long term impact on both the public health and the economy. As AR has been steadily increasing since the 1940’s, causing a long term impact on the worldwide health and economy systems, it can rather be categorized as an endemic (MacIntyre and Bui, 2017). Whether an endemic rate will maintain, increase or decrease is unpredictable, but the sources of any endemic will always stay the same (Bloom and Cadarette, 2019), and this renders a very positive starting point for tackling the whole AR phenomenon, as the roots of an endemic are mostly always known.

In the US alone over 2 million people acquire a nosocomial infection each year, with around 70% of such infections being caused by MDR-strains (Memoli et al., 2008). On the other hand, up to 87% of farm animals have been described to be resistant to amoxicillin and oxytetracyclin, 85% to trimethoprim, 81% to neomycin and 66% to flumequine, among others (van den Bogaard et al., 2001). Moreover, 90% of seawater-originated bacteria are resistant to at least one antibiotic, and up to 20% are resistant to at least five, while antibiotic traces can be detected in 52% of fish samples (Watts et al., 2017). Finally, different r genes can be found on 27.1% of regular sewage bacteria (Parnanen et al., 2019), while the richness and abundance of r genes and MDR bacteria are vastly increased in airplane sewages (Hess et al., 2019), presumably because of HGT among bacteria that are native from different parts of the world.

Following this line of thought, farms, sewages as well as human and veterinary hospitals are critical sources for the AR endemic, so that action in these sectors must be undertaken in order to control such problem. Although social control measures have been applied as means to limit antibiotic usage and accessibility by the general population, such measures are yet to produce the desired effect of lowering the rate of AR dissemination (Uchil et al., 2014). In this way, an interdisciplinary approach must be conceived, and we think that the strategies proposed in such plan must be aimed at the control of resistant microbes in the aforementioned sectors through the use of newer technologies that difficult AR-bacterial spread and development.

Furthermore, AR might be a problem for the end users of antibiotics, nonetheless, the control of the whole phenomenon may not happen at the patient’s treatment level because the aforementioned hot points are reservoirs for MDR-bacteria, where these pathogens may persist even after the treatment of infected patients with new effective drugs. Rather, ecological control in the aforementioned areas might be a complementary strategy in order to prevent such infections in the first place, and to reduce the likelihood for the emergence of new AR-bacteria. However, to our notice there are no biotechnological measures or devices in commercial use in order to cope with this awn of the problem. As such, we think that devices with long-lasting antimicrobial properties, such as those that can be fabricated through NP-based coatings, may be of considerable utility for such application.

## Fighting Fire With Fire: Nanotechnology to Combat Antibiotic Resistance

Professor Norio Taniguchi from Tokyo Science University introduced the term “Nanotechnology” in the year 1974, and in this word he described precision manufacturing of materials at the nanometer level. Later, Professor Richard P. Feynman, who was a physicist, gave the concept Nanotechnology in his lecture “There’s plenty of room at the bottom” (Rai et al., 2009). In general we used the word “nano” to indicate that something has a size of one billionth of a meter or 10^–9^, with a word that is synonymous to “dwarf”. Thus, the term “nanoparticle” refers to clusters of atoms in the size range of 1–100 nm (Rai et al., 2009), and interestingly, some of them possess reduced production costs (Huh and Kwon, 2011).

Thus, it may seem that ANPs are a recent development, but indeed they have been investigated (Lea, 1889), and even commercially available, for the last 120 years. To our notice, their first commercial application was an antiseptic named “Collargol”, which was made of colloidal silver (Fortescue-Brickdale, 1903) that contained silver particles with an average size of 10 nm. Even when the “nano” nomenclature was not used at that time, the increased efficiency of the nano-scaled silver was recognized (Nowack et al., 2011).

Nowadays, many reports have given details about the antimicrobial properties of some NPs, and even activity against AR-bacteria (Andrade et al., 2013). These nanomaterials (NMs) are divided in mainly two groups, according to their chemistry, such being metallic and non-metallic. A vast variety of NPs exist, but the most important in terms of antibacterial properties are: iron oxide (Fe_3_O_4_NPs), zinc oxide (ZnONPs), copper oxide (CuONPs), titanium dioxide (TiO_2_NPs), silver (AgNPs), gold (AuNPs) graphene oxide (GrONPs), and reduced graphene (rGrNPs) NPs (Yousefi et al., 2017).

Along with Alexander Fleming, Paul Ehrlich was one of the pioneers of the so called “antibiotic era.” He was a great promoter of the idea that chemical compounds “able to exert their full action exclusively on the parasite harbored within the organism” could be synthesized, leading to the concept of antibiotics as “magic bullets” to kill pathogens exclusively, without damaging the host. Despite antibiotic’s success in their specific task, such bullets have attacked bacteria through only one mechanism at a time, which may have favored AR resistance, as altering the bullet’s target has been a common bacterial strategy to escape antibiotic’s action. On the other hand, the aforementioned NPs do not bind to a specific receptor on the bacterial cell, rather, they have different mechanisms of action, and this characteristic complicates the development of resistance by bacteria, and broadens the spectrum of each kind of NP.

In general, several of the main toxicological effects of NPs occur by direct contact with the bacterial cell surface (Wang et al., 2017), which makes important to understand their properties. Gram-positive bacteria have a thick layer of peptidoglycan and negatively charged teichoic acids (phosphate groups), while Gram-negative bacteria have a thin layer of peptidoglycan associated to a phospholipid outer membrane with lipopolysaccharides that are also negatively charged. Such structural features are important because the primary interaction of metal-based NPs with bacteria is mediated by electrostatic attraction between opposite charges, which leads to a strong bond that triggers their biologically relevant mechanisms of action, which are different for each metal (Guzman et al., 2012). Moreover, structural factors of the NPs influence their antibacterial activity. For example, its size (a smaller size enhances their surface area, which improves their association with cell wall or cell membrane) (Seil and Webster, 2012; Agnihotri et al., 2014), morphology (shapes that augment their surface enhance their function) and dose (the higher the concentration the greater the result) (Breunig et al., 2008).

For the purposes of this article, we chose three “prototypical” kinds of NPs: silver, gold and graphene. The first one being a metallic NP that has a strong standalone action over bacteria, the second one being another metallic NP that lacks such standalone activity, and the last one being a non-metallic type of NP that has a strong antibacterial activity on its own, as we will discuss the potential applications of these three kinds of NPs in the development of antimicrobial contraptions.

## Silver Nanoparticles

Silver has been known as a metal from as early as 4,000 BCE and it was used for making coins, utensils, containers, jewelry, and other objects (Gharpure et al., 2020). Moreover, such element has been used for a long time for the treatment of ulcers (Alexander, 2009), infections, burns and chronic wounds, as well as to make water potable, nonetheless, its true potential in medicine was unknown at that date as this metal was yet to be used in its nanometric size, which enhances its antimicrobial potential. In recent years the AgNPs have been used as disinfectants, but their use as drugs has been limited as AgNPs can produce *agyrosis*, a condition that causes a blue-gray or purple coloration on the skin, mucosal membranes and conjunctiva (Rai et al., 2009).

The AgNPs have also been used in many applications on the physical and chemical sciences, being synthesized in different morphologies like spherical, conical, rod-like wires, etc., but these applications are beyond the scope of this review (Syafiuddin et al., 2017). On the other hand, AgNP uses in biological sciences to combat AR bacteria in products like toothpaste, deodorants, food packaging, cosmetics and water filters are, on the other hand, of great interest for the present discussion (Gharpure et al., 2020).

Different methods for synthesizing AgNPs have been used, and are based on physical, chemical or biological approaches (Song and Kim, 2009). Physical (top-down) and chemical techniques (bottom-up), in general, yield the highest efficiency and production rates, but these methods involve use of toxic and expensive chemicals (Gharpure et al., 2019). For this situation the green synthesis has been proposed as an eco-friendly, unexpensive, reliable, fast, biocompatible, and clean alternative (Gharpure et al., 2020).

Disregarding their synthesis method, AgNPs have been shown to inhibit a wide range of HIV-1 strains, both Gram positive and Gram negative bacteria and parasites such as *Leishmania tropica, Entamoeba histolytica, Cryptosporidium parvum, Giardia lamblia, Fasciola* spp., *Plasmodium* spp., and *Toxoplasma gondii*, whether resistant to antibiotics or not, therefore they are considered to possess a broad spectrum of action (Table 1).

**TABLE 1:** Mechanisms of action and spectrum of activity of nanoparticles.

**Nanoparticle**	**Action Mechanism**	**Spectrum**	**References**
AgNPs	• Alteration of membrane permeability. • Binding to membrane proteins (respiratory chain proteins, transport proteins) and interfering with cell division and ion transport processes. • Inhibition of transcription, translation and protein synthesis. • Generation of ROS causing a direct damage on DNA.	*Leishmania tropica Entamoeba histolytica Cryptosporidium parvum Giardia lamblia Fasciola spp. Plasmodium spp. Toxoplasma gondii Escherichia coli Pseudomonas aeruginosa*	Ivask et al., 2014; Habimana et al., 2018; Ahmad et al., 2013; Eigler and Hirsch, 2014; Soleymani et al., 2016; Amrollahi-Sharifabadi et al., 2018
AuNPs	• Interfering with protein and ATP synthesis. • Modifiying membrane potential. • Inhibition of H^+^ transport	*Escherichia coli Pseudomonas aeruginosa Salmonella typhi Bacillus subtilis Staphylococcus aureus Klebsiella pneumoniae Candida albicans Candida glabrata*	Khezerlou et al., 2018; Kumar et al., 2019
Graphene	• Disruption of cell membrane by nano-blades. • Trapping of the bacterial membrane. • Destructive extraction of membrane lipids. • Oxidative stress which interfere with bacterial metabolism.	*Escherichia coli Staphylococcus aureus Pseudmonoas aeruginosa Bacillus subtilis Enterococcus faecalis Staphylococcus epidermidis*	Mu et al., 2016; Barbero et al., 2017; Ahsan et al., 2018; Kang et al., 2019

**FIGURE 1:**
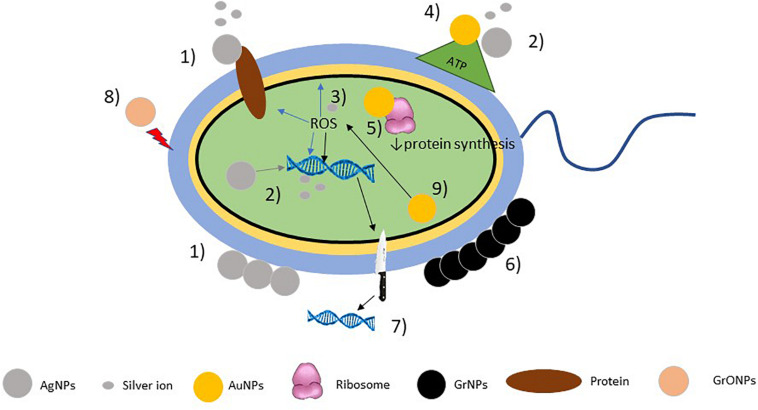
Mechanisms of action of nanopaticles. AgNPs 1) bind to cell membrane neutralizing its charge, thus altering its permeability and affecting membrane transport and respiratory chain proteins; 2) release silver ions affecting genetic expression and ATP synthesis; 3) and generate ROS affecting DNA and cell membrane. AuNPs 4) inhibit ATP synthase; 5) inhibit tRNA binding to the ribosome; GrNPs 6) entrapment of the bacterial membrane; 7) form nano-blades that cut the cell membrane and destroy DNA; GrONPs 8) destroy the bacterial cell membrane; and 9) ROS-dependent and ROS-independent oxidative stress.

The biological effects of AgNPs is dependent on the following mechanisms: (a) the silver NPs binding to the cell wall altering its permeability, which was demonstrated in studies conducted on gram negative bacteria such as *Escherichia coli* and *Pseudomonas aeruginosa*, where the neutralization of the bacterial surface charge altered the cell membrane’s permeability (Ramalingam et al., 2016) and in *E. coli*, where scanning and transmission electron microscopy studies demonstrated that these NPs are able to produce pits in the cell walls, where the AgNPs accumulated (Sondi and Salopek-Sondi, 2004); (b) AgNPs are able to bind to membrane transport and respiratory chain proteins, thus interfering with the cell division and ion transport processes, ultimately leading to bacterial death (Ivask et al., 2014); (c) their nanometric size (1–10 nm) allows them to penetrate into bacteria affecting intracellular process leading to inhibition of transcription, translation and protein synthesis (Rai et al., 2009; Reidy et al., 2013). This mechanism is explained by the release of silver ions which could be generated and introduced by oxidative dissolution of AgNPs in the presence of oxygen (Reidy et al., 2013). Such ions with positive charge have high affinity with the negatively charged sulfhydryl, phosphate and carbonyl groups of the bacterial cell membrane, proteins and DNA bases (Rai et al., 2009; Xiu et al., 2012; Chung et al., 2016) and upon contact lead to their alteration. Also, they are able to impair the transport and release of potassium ions and to block the synthesis of ATP in the bacterial cells (Hsueh et al., 2015). It has been shown that smaller NPs not only have an enhanced reactivity, but can also release more of these ions to an enhanced antimicrobial activity (Jung et al., 2008). Additionally, (d) AgNPs are able to dissipate the proton motive force of bacterial enzymes and transporters, thus causing an accumulation of envelope protein precursors and causing a destabilization of the outer membrane, collapse of its potential and depletion of intracellular ATP levels (Lok et al., 2006). Finally, (e) AgNP and silver ions lead bacteria to generate reactive oxygen species (ROS) such as superoxide anions, hydrogen peroxide, and hydroxyl radical (Manikprabhu and Lingappa, 2013), as well as malondialdehyde, protein carbonyl content, and nitric oxide (Gurunathan et al., 2018), which on increased concentrations cause a direct damage to bacterial DNA causing chain breakage, cytosine, adenine and guanine deamination, lipid peroxidation and also block the activation of the enzymatic DNA repair (Figure 1).

As a side note, AgNPs also possess anti-HIV activity, by inhibiting the initial phase of fusion and entry of HIV-1 mediated by the gp120-CD4 interaction in a dose-dependent manner. Also AgNPs inhibit the first phase of the replication cycle at the same place where the antiretroviral enfurtivide acts. Moreover, AgNPs also have antiparasite activity, as they enter directly in the protoctist cell membrane through ion channels, transport proteins or by endocytosis, and then they inhibit promastigote proliferation, damage DNA and generate ROS (Khezerlou et al., 2018).

### Gold Nanoparticles

Gold has been known to man since the pre-history, and has been used as a coin since 3100 BCE. It is considered as a noble metal due to its inertness towards other atoms and molecules, which makes for its apparent lack of cytotoxicity to human cells and a great overall biocompatibility (Gharpure et al., 2020). Its innate properties have been exploited for a plethora of biomedical applications like targeted drug delivery, biosensors, optical imaging, as well as detection of microorganisms (Gharpure et al., 2020). Many studies have also shown the antimicrobial properties of this type of NPs, especially in many pathogenic species (Dizaj et al., 2014). AuNPs can be fabricated using “top-down” as well as “bottom-up” methods, however, green synthesis has also been favored for its environmental friendliness (Shah et al., 2014).

Gold nanoparticles (AuNPs) are effective against many strains of bacteria and fungi such as *E. coli, P. aeruginosa, Salmonella typhi, Bacillus subtilis, Candida albicans, Candida glabrata*, and some major pathogenic bacteria like *S. aureus* and *K. pneumoniae* (Table 1), including AR-strains. Unlike AgNPs that act alone, AuNPs act better in association with antibiotics, vaccines and antibodies, as they enhance their intracellular transportation, and diffusion, but alone, their antimicrobial properties are mainly dependent on its ability to interfere in protein synthesis by the inhibition of the tRNA’s ability to bind the ribosome (Cui et al., 2012), to inhibit ATP-synthesis by the inhibition of ATP-ase and damaging the microbial cell membrane (Nisar et al., 2019), as well as their ability to modify the membrane potential (Gharpure et al., 2020). Additionally, these NPs seem to disrupt biofilms, although this activity is greatly enhanced when AuNPs are conjugated with proteinase-K (Habimana et al., 2018). On the other hand, their antifungal activity is due to the inhibition of H^+^-ATPase at the plasma membrane, inhibiting the H^+^ transport chain (Ahmad et al., 2013; Figure 1).

### Graphene Nanoparticles

Graphene (Gr) is an allotrope of carbon that consists of a single layer of atoms in a two dimensional lattice, it is the basic structural element of other allotropes, including graphite, charcoal, carbon nanotubes and fullerenes (Eigler and Hirsch, 2014). Since its discovery by Prof. Andre Geim and Prof. Kostya Novoselovt in 2004 through micromechanical exfoliation of graphite (Zhang et al., 2012), Gr has been regarded as the strongest material in the world, and its extreme elasticity and conductivity have also been recognized, placing it as a wonder material with many applications in biomedicine for the diagnostic and treatment of diseases (Amrollahi-Sharifabadi et al., 2018). Moreover, Gr can also be synthesized in an oxidized form to yield graphene oxide (GrO), which possesses chemically reactive oxygen groups such as hydroxyl, carboxylic acid, and epoxy which enhance its reactivity (Soleymani et al., 2016).

Because of their two-dimensional structure Gr and GrO have a large specific surface area that enhances their reactivity and gives them some of their interesting antibacterial properties (Hasanzadeh et al., 2016), but also their size, oxidation level, functionalization, or electronic structure are able to modulate their activity (Perreault et al., 2015). Their proposed mechanisms of action involve both physical and chemical activities, where the physical modes of action are the most extensively found in experimental settings, such as direct damage by contact of the bacterial membranes with the sharp edges of graphene and wrapping of the bacteria. On the other hand, the chemical modes involve oxidative stress by ROS generation and charge transfer (Kumar et al., 2019).

For instance, on the physical damaging strategies of both pristine (Tu et al., 2013) and oxidized (Hui et al., 2014) graphene Tu et al. (2013) observed the degradation on the inner and outer cell membranes of *E. coli*, as well as the reduction of the colony’s viability in relation to the ability of Gr to penetrate such structures to extract its phospholipid contents. Such damage resemble the formation of pores on the membrane, and it has been further suggested that the density of Gr edges, rather than the size or shape of such structures, is the main factor enhancing its antibacterial ability (Pham et al., 2015). Moreover, GrNPs are able to cause membrane stress by direct contact with the sharp edges of their nanosheets, which disrupts and damages cell membranes, thus leading to the release of intracellular contents in a mechanism of action termed as “nano-blade.” Also, GrNPs are able to aggregate in the surroundings of the bacterial cells to trap bacteria in Gr-based cages, isolating them from the nutrients from the environment and thus leading to bacterial cell death (Liu et al., 2011; Zou X et al., 2016). Both GrNPs and GrONPs are able to produce ROS-dependent and ROS-independent (derived from the oxidation of glutathione) oxidative stress, which interfere with bacterial metabolism and disrupt cellular functions (Liu et al., 2011; Zou X. et al., 2016), while this effect is stronger for GrO (Liu et al., 2011; Figure 1). Both materials have exhibited good antibacterial properties against many bacteria, to include *E. coli, S. aureus, P. aeruginosa, B. subtilis, Enterococcus faecalis*, and *S. epidermidis* (Pham et al., 2015; Table 1), many of which are AR strains.

As such, both materials have been used to disperse, stabilize and deliver drugs, like antibiotics, but such materials also exhibit good antimicrobial properties on their own. Such antibacterial activity, coupled with Gr and GrO’s flexibility and resistance, has led to the design of antibacterial packaging for food, water treatment devices, wound dressings and disinfectants (Ji et al., 2016).

### Summary of Antimicrobial NPs’ Mechanisms of Action

As mentioned above, NPs have more than one mode of action over bacteria, which enhances their potential to damage different prokaryotic structures at the same time, thus augmenting their overall antibacterial activity. However, it is not clear if all the reported effects for each NP occur with every bacterial species, nor if all of the antimicrobial mechanisms reported for a particular chemical NP type are the same when the size and shape of the NPs is different. In such panorama, comparative studies to determine what size, shape and chemical type of NPs possess the strongest antibacterial activity, and over what bacterial species do they exert these effects are needed in order to determine the best ways to use these agents. Moreover, this kinds of studies may serve to better assess if resistance to NPs at optimal doses is possible, and may also serve to focus studies about their toxicity in a more efficient way, as these topics have been an intense matter of discussion over the years, finding conflicting results.

Moreover, much of these assays have been carried out *in vitro*, while emerging properties in living systems have not been taking into proper account for the assessment of NPs’ antimicrobial activity. For instance, it is known that upon contact with NPs proteins tend to aggregate to the NPs’ surface to constitute a structure known as “biomolecular corona,” which alters various NP’s properties, like targets, toxicity, immune-modulating properties and interaction with cellular structures (Barbero et al., 2017; Ahsan et al., 2018). This phenomenon could also alter the way that NPs interact with bacteria, changing entirely their antimicrobial properties when they are used as drugs. Moreover, antimicrobial NPs do combat intracellular bacteria in co-cultures of bacteria and host cells, but with decreased efficiency in comparison with extracellular bacterial infections (Kang et al., 2019). In such cases the NPs’ antibacterial ability was enhanced through conjugation with antibiotics, as NPs worked as vehicles for the antibiotics and synergized with them (Mu et al., 2016). Nonetheless, it is still unknown whether NPs may be able to combat intracellular infections in an *in vivo* setting, in which dose and with what efficiency. Thus, as happens with many emerging nano- and bio-technologies, much research is needed to complete our understanding of NPs’ properties, and *in vivo* acquired data is of paramount importance in this case.

## Bacterial Resistance to Nanoparticles: Is it Possible?

As NPs act on bacteria by multiple mechanisms at the same time, the development of resistance to them should come from several accumulated mutations as a mean to modify some, or all, of the NPs’ bacterial targets. In this way, some authors think that such phenomenon is highly unlikely (Baptista et al., 2018), but others have detected some degree of resistance mainly to metallic NPs, as thoroughly reviewed in (Nino-Martinez et al., 2019). To date, electrostatic repulsion, ion efflux pumps (Yang et al., 2012), production of protective extracellular matrixes (Zhang et al., 2018), mutations (Graves et al., 2015), and biofilm adaptations have been detected among bacteria in order to resist the attack of such NPs (Agnihotri et al., 2014).

Interestingly, the detection of NP-resistance (NPR) has been made in experiments where bacteria have been cultured in mediums with low metallic-NP concentration, but to our knowledge, there is no data about NPR occurring in cultures with the minimum inhibitory concentrations for each type of NP. Moreover, NPR may be countered easily by many strategies, to include the regulation of NP’s size as a reduction of their dimensions leads to an increased surface/volume ratio and thus to enhanced antimicrobial activity (Agnihotri et al., 2014), which reduces the possibility of bacterial survival and development of resistance through mutations (Graves et al., 2015) and development of ion efflux pumps (Yang et al., 2012). Also, agents like simvastatin and pomegranate rind extract have been demonstrated to inhibit the extracellular aggregation of metallic-NPs that occurs through the bacterial production of an extracellular matrix and by flagellin expression (Panacek et al., 2018; Cui et al., 2020). In this way, a correct design of the NP-based therapies or devices may not induce the development of NPR.

To our knowledge, there is no data regarding the development of resistance against GrNPs or GrONPs, but excellent research by Guo and Zhang (2017) and Zou W. et al. (2016) have shown that GrONPs are able to damage r genes-carrying plasmids and lower the abundance of the r genes *sulI* and *intI*, which suggests that resistance to GrONP is unlikely to happen. Furthermore, such investigations suggest the basis of a strategy to halt the HGT of genetic resistance determinants, which may be of use in the dampening of the whole phenomenon of AR.

On the other hand, either bimetallic NP (Gopinath et al., 2016) or GO-Ag nanocomposites (de Moraes et al., 2015) have shown increased antibacterial activity when compared to monometallic or non-composite NPs, but research regarding their ability to halt r genes or about the bacterial ability to develop resistance against such materials is lacking, but their increased antimicrobial abilities appear as promising alternatives to avoid resistance development.

## Translation to the Clinical Setting: Are Nanoparticles Safe to Use?

The prototypical path to introduce new drugs into the clinical setting requires vast preclinical testing of the candidate products to produce information about their efficacy and safety. Drugs that appear to be safe in animal models and human cell studies, may then be evaluated for their further study in phase 1 clinical trials, where safety and dosage in human patients will be assayed in no more than 100 volunteers, in a period that typically lasts for ≈3 years. If they are found to be safe, further tests of effectiveness, side effects and adverse reactions of the long term will be performed in phase 2 and 3 clinical trials in a period that may last up to ≈13 additional years. Notwithstanding, all new products must prove to be safe in the preclinical setting in order to be approved for phase 1 trials (Lipsky and Sharp, 2001). Accordingly, we and other researchers (Min et al., 2015) think that the translation of some applications of the antimicrobial NPs into the clinical setting may be difficult and/or greatly delayed as there is not much consistency on their safety profile, as contrasting results have been shown on several *in vitro* and *in vivo* models.

For instance, AgNPs have shown good *in vitro* compatibility with human and murine erythrocytes (Hossain et al., 2019), but they do exhibit toxicity to peripheral blood mononuclear cells (PBMCs; Shin et al., 2007). Moreover, *in vivo* studies on zebra fish made by Bao and colleagues show that AgNPs induce pathological changes on growth indices, oxidative/antioxidative status, neural signaling, Na/K-ATPase function, and antioxidant system in the intestine, but not in the liver, with male zebrafish being more sensitive than female (Bao et al., 2020). Despite the absence of liver damage on zebra fish observed by Bao, further studies on liver damage show that although murine healthy livers do not appear to suffer much damage by AgNPs, these NPs appear to enhance ethanol-induced inflammatory damage in such organ (Kermanizadeh et al., 2017). Moreover, although functionalization of AgNPs with either polyvinylpyrrolidone (PVP) or citrate appears to enhance these NP’s antimicrobial activity, it enhances their potential to induce cellular toxicity as well as inflammatory and oxidative stress on murine lungs, thus leading to mild pulmonary fibrosis (Wang et al., 2014). Together, these results suggest that AgNPs may possess a certain degree of toxicity that may be impossible to overcome, but on the other hand Rezvani et al. (2019) suggest that AgNPs may be safe on certain limited doses. Nonetheless, a reduction on these NP’s doses may lead to NPR (Graves et al., 2015), in such a way that more research is needed in order to best describe a safe and effective way of using AgNPs as drugs.

Although AuNPs are generally thought to be safe, they have been shown to accumulate in the intestine, kidney, liver, spleen, and colon, while also being aggregated in the nucleus of hepatocytes and colonic cells, where they produce DNA damage, with smaller NPs exhibiting more toxicity (Lopez-Chaves et al., 2018). Moreover, such NPs have also been demonstrated to induce pulmonary inflammation, disregarding their size (Gosens et al., 2010).

Moreover, non-metallic NPs have also shown a certain degree of toxicity as chicken embryos that were injected with diamond, graphite, Gr and GrO NPs into the egg albumin showed decreased survival, but there were no differences on body and organ weight, red blood-cell morphology, blood serum biochemical parameters, and oxidative damage in the surviving embryos in comparison with the placebo group (Kurantowicz et al., 2017). Also, upon inhalation GO nanosheets induce pores in the pulmonary surfactant film, thus altering its ultrastructure and biophysical properties (Hu et al., 2015). Furthermore, in the graphene family of NPs cytotoxicity dependent upon physical destruction of cell components, oxidative stress, DNA damage, inflammatory response, apoptosis, autophagy, and necrosis have been detected in many different models, as thoroughly reviewed in (Ou et al., 2016). Nonetheless, in other *in vivo* studies pathological changes in weight gain, hematological and biochemical parameters have only been detected in rats that received high GrONP-doses (500 mg/kg), but not lower doses (50 or 150 mg/kg) (Amrollahi-Sharifabadi et al., 2018).

Despite the aforementioned evidence, which seems to render antimicrobial NPs mostly as toxic substances, controversy has arisen in regards to this topic, as some authors report a safe profile for NPs while other researchers find cues of their toxicity. This controversy may arise from the fact that NP are not created equally and have different properties. Aside from the dose (the larger the dose, the more toxic it is) (Graham et al., 2017), the size (the smaller the NPs the more toxic that they are) (Pan et al., 2007; Kim et al., 2012), shape (the most surface they have, the more toxic they are) (Wang et al., 2008; Nam and An, 2019), surface charge and chemistry of NPs (Zoroddu et al., 2014) also relate to increased NP toxicity, and may change the NP’s absorption properties, accumulation, distribution and elimination, thus complicating the study of their toxicity. On the other hand, surface functionalization of such materials has been proposed as an alternative to improve biocompatibility (Subbiah et al., 2010), but research has shown that in some cases (Wang et al., 2014; Zhang et al., 2016) this strategy enhances toxicity.

Lastly, most of the assessments for NPs’ toxicity comes from *in vitro*-performed experiments, where emerging properties of living systems are neglected. In the setting of living beings, at least three physiological phenomena could impact the final toxicological performance of NPs, which are: (a) the fact that cells possess different shapes when they are part of a three dimensional tissue, in comparison to those that are cultivated in two dimension culture plates. This phenomena has only recently been recognized as a factor determining NP uptake and toxicity (Farvadi et al., 2018). Secondly, (b) the formation of the “biomolecular corona” that happens when NPs come in contact with biomolecules, such as proteins (Ahsan et al., 2018) which could alter their toxicological properties significantly. And lastly, (c) the fact that many bacterial infections are intracellular, and that although NPs are able to combat intracellular infections, they do so with decreased efficiency over extracellular colonization (Kang et al., 2019), which may impact the efficiency of the dose.

In this way the study of nanotoxicology becomes increasingly complicated with NP diversity and with the interaction with the host, which may explain the emergence of the aforementioned controversy regarding the safety of NPs for use in medicine. Nonetheless, much research is needed in a standardized manner to accurately assess the toxicity of NPs (Zoroddu et al., 2014; Hofmann-Amtenbrink et al., 2015) in different doses and routes of administration, and then get to know their potential in the clinical setting, which may be of enhanced importance in order to fully explode this promising technology.

Taken together, these data suggest that antimicrobial NPs may not be safe for their use as drugs, or at least, that much knowledge is needed in order to establish the right windows for dosage, administration routes, functionalization strategies and physical properties for their safe use. Thus, this knowledge, or lack of knowledge may halt NPs’ approval for clinical use. In such panorama, more research is needed in order to enhance our knowledge about the modulation of NPs’ toxicity and activity trough functionalization and regulation of NP’s dose, size, shape, and chemistry in order to fully exploit their potential, but also research about alternative uses for NPs that do not involve administration into the human body are critical, as a strategy to exploit their potential as soon as possible and dampen their toxicity at the same time.

One could argue that the last decade has witnessed a rise in nanomedicine, with many kinds of NPs being investigated and applied into the clinics, but the nano-drugs that have been approved for their clinical use, or that are actually being investigated in clinical trials are mainly anti-tumoral agents, supplements, imaging contrasts and agents, drug-delivery vehicles, anesthetics (Anselmo and Mitragotri, 2019), and even as biosensors for the detection of infectious diseases (Colino and Millán, 2018), but little to no advances have been made in regards to ANPs into their clinical application, which reflects the importance of this window of opportunity.

## Antimicrobial Nanoparticle-Based Medical Devices: Towards the Ecologic Control of MDR Bacteria

As stated above, many experiments have rendered antimicrobial NPs as good alternatives to fight AR-bacteria, and some types of NPs may even be able to avoid resistance development, but the use of such technology as drug alternatives may be halted due to the numerous evidences about its toxicity. Nonetheless, as promising research shows, antimicrobial NPs may have an alternative niche in the combat of AR pathogens trough NP-based medical devices, where the NPs are not administrated into the body, or have a minimal contact with it, but can be used as barriers in endemic zones for AR to control the spread of AR-bacteria. In this way, such contraptions may exert ecological control of such pathogens while avoiding toxicity to the human body.

**FIGURE 2:**
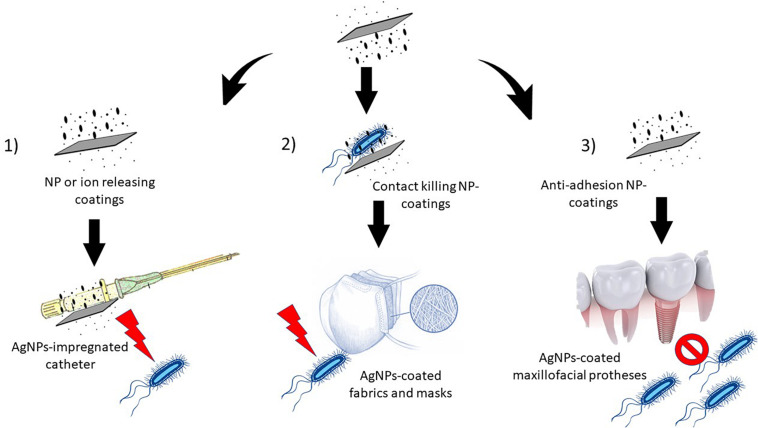
Types of nanoparticle-based devices. 1) The NP impregnated devices release NPs or ions over time, thus killing bacteria (e.g., AgNP-impregnated catheters), 2) the coating actively kills bacteria by contact (e.g., AgNP-coated fabrics and surgical masks), and 3) biofilm adhesion is inhibited by the coating (e.g., maxillofacial prostheses and dentifrices).

As stated in the above sections, hospitals are considered endemic zones for AR-bacteria and AR development, and within a hospital, textile fabrics have been described as important reservoirs and fomites (Neely, 2000; Neely and Maley, 2000; Pilonetto et al., 2004). Moreover, other biomedical devices that come in contact with the patients, like catheters and wound dressings, are also important vectors for the transmission and dissemination of nosocomial bacteria. As such, the prevention of microorganism colonization and consequent biofilm formation in these devices could limit AR-development (Palmieri et al., 2016). Three major types of antimicrobial coatings have been designed to accomplish this task: (i) the ones that work through antibacterial NP release, others that work by (ii) contact-killing, and finally (iii) by halting bacterial adhesion. In the first case, the coating is loaded with a drug that is released over time by diffusion or erosion, and because the release is local and gradual, it limits systemic effects. In the second case, bacteria are directly killed by contact with the coated portion of the device, and in the third, biofilm adhesion becomes impaired (Figure 2). In every case, the potential harmful effects of such coatings are limited to the contact area and its near surroundings, so that they appear to be safer than NP-based drug candidates (Palmieri et al., 2016).

Following this line of thought, Duran et al. (2007) developed AgNP-coated cotton fabrics that demonstrated a significant antibacterial activity against *S. aureus*, while Li et al. (2006) developed titanium dioxide and silver-nanoparticle coated surgical masks that killed 100% of both the *E. coli* and *S. aureus* bacteria that were incubated in such masks, without producing skin irritation on their volunteered wearers.

On the other hand, AgNP-coated wound dressings have an enhanced ability to prevent bacterial colonization and biofilm formation at wound sites while promoting tissue regeneration, and thus have been rendered as useful in the treatment of extended wounds and burns (Halstead et al., 2015). Moreover, nanocomposites of silver and graphene with a ratio of 5:1 exhibit stronger antimicrobial and wound healing abilities than other hydrogels, while exhibiting low toxicity as evidenced by an MTT (3-(4,5-Dimethyldiazol-2-yl)-2,5-Diphenyl Tetrazolium Bromide) assay (Fan et al., 2014), suggesting that nanocomposites may have an enhanced function over simple NPs, perhaps as a function of the addition of two different sets of action mechanisms (Table 2).

**TABLE 2:** Nanoparticle-based antimicrobial devices: uses and possible future applications.

**Devices**	**Nanomaterial**	**Advantage**	**Shown Application**	**Possible further uses**
Cotton fabrics	AgNPs	Significant antibacterial activity against *S. aureus*	Antimicrobial textile fabrics	Surgical uniforms, surgical fields, bedding and scrubs for hospital and veterinary use Bedding materials, pen and cage protections in farms
Surgical masks	Titanium dioxide and silver-nanocomposite	Kills 100% of both, *E. coli* and *S. aureus* without skin irritation	Surgical masks	Air filters coupled to air conditioning devices
Coated wound dressings	AgNPs	Prevent bacterial colonization and biofilm formation at wound sites while promoting tissue regeneration	Treatment of extended wounds and burns	May be used for veterinary applications
Coated catheters	AgNPs	Dampening of catheter-produced bacteremia	Reduction in bacterial colonies	Building components for hemodialyzers, blood oxygenators, and arterial filters, among other biomedical devices. Hoses to transport water into farms. Sewages.
Coated maxillofacial silicone prostheses	AgNPs	Good biocompatibility and antimicrobial actions	Antibacterial maxillofacial prostheses	Sealing materials for building farms and hospitals, sewage joints. Base materials for water bottles and filters
NP-coated polyurethane and polycarbonate	GrO	Good biocompatibility and antimicrobial actions	None	Pipes, toilets, hoses and panels
Dentifrice	Nano-silver fluoride	Effectively kills *S. mutans*, prevents bacterial adhesion to teeth and control teeth acidification	Paste for mouth washing and caries prevention	Abrasive cleaners for farms, hospitals and sewages
Water disinfecting filters	Ceramic membranes coated with Ag/GrO nanocomposites	Eliminates *E.coli* and *S. aureus* in water	Water disinfecting	Disinfection of water for human and animal consumption and of sewage water
Graphene coated titanium	Electrodeposition of graphene on titanium	Antibacterial activity against *S. aureus* and *E. coli*. Compatibility with peripheral blood mononuclear cells	Antimicrobial Gr-based coating of a metal	Surgical materials. Posts and plates for building hospital beds, pens and cages.

This technologies could be extrapolated to their use in surgical fields, intensive care unit (ICU) bedding and scrubs for its use in both human and animal hospitals; but also could be used to produce bedding material and protections for pens and cages, or even to produce air filters to control AR-bacteria spreading in farms (Table 2).

Furthermore, in the late 90’s nano-silver coated catheters were clinically investigated for their potential to reduce hospital acquired bacteremia with disappointing results (Darouiche et al., 1999; Antonelli et al., 2012), nonetheless further preclinical investigations showed that a supercritical carbon dioxide impregnation method, as opposed to coating, showed an increased release of silver ions that could lead to enhanced antibacterial actions (Furno et al., 2004; Table 2). In regard to this technology, a thorough investigation on whether the released silver ions could potentially produce toxic effects or not should also be critically evaluated, but the hemocompatibility that AgNPs have shown (Hossain et al., 2019) may suggest a safe profile for such application. On the other hand, AgNP-coated maxillofacial silicone prostheses have suggested good antimicrobial abilities and good biocompatibility measured by fibroblast viability after exposition (Meran et al., 2017; Table 2), enhancing the notion that polymeric materials that are coated with NPs may be safe for use.

Moreover, polymers like polyurethane and polycarbonate are widely used in hospitals for residue disposal and to build masks, physical barriers, hemodialyzers, drug delivery carriers, blood oxygenators, and arterial filters, among other biomedical devices. In this way, NP-based antimicrobial coatings for such materials may also help to control AR bacteria, not only by building devices that would be used in close contact with the medical staff and patients, but also by serving as building blocks for critical points of a hospital’s facilities. Consequently, both GrO reinforced polyurethane (An et al., 2013) and polycarbonate (Mahendran et al., 2016) have been tested for potential antibacterial effects, finding good results on such ability and on their safety profiles (Mejias Carpio et al., 2012). Also, AgCl-TiO_2_ nanocomposite was demonstrated to be an excellent matrix that released silver ions to the surroundings to inhibit biofilm formation (Naik and Kowshik, 2014). These findings could be extrapolated to AR-endemic zones, like hospitals, farms and sewages, as these materials may be used in these facilities as building blocks for pipes, hoses and panels (Table 2).

Most of the research regarding the coating of biomedical devices with antimicrobial NPs has been aimed at polymers, but metallic surfaces can also be coated with such substances. An example being the electrodeposition of graphene on titanium, which facilitates hydroxyapatite aggregation and possesses interesting antibacterial abilities against *S. aureus* and *E. coli*, while being compatible with PBMCs (Janković et al., 2015) (Table 2). In this way, we think that this development may help to fasten prostheses with an enhanced level of security, or even to augment the sterility of scalpels, among other applications, but research about NP-coatings on other materials that are more common in hospitals, farms or sewages, like aluminum or steel, may enhance the reach of such applications in the war against AR bacteria.

On the other hand, a dentifrice containing nano-silver fluoride was shown to effectively kill *S. mutans*, prevent bacterial adhesion to teeth and to control teeth acidification significantly better than sodium fluoride-based dentifrices, thus protecting the tooth enamel in an enhanced way and suggesting a potential effectiveness to prevent caries (Teixeira et al., 2018). While the authors of the study did not evaluated the toxicity of such dentifrice, we think that the complete process of mouth washing may be able to reduce the exposition of the users to the dentifrice’s nano-compounds. Nonetheless, the absorption potential of the mucosal tissues of the mouth may enhance the infusion of such NPs into the patient’s system, so that studies about this kind of application should be thoroughly conducted.

As sewage water is another endemic zone for AR-development, water disinfecting devices may be another route to cope with the ongoing problem of AR bacteria. Following such line of thought, water treatment devices that are based on ceramic membranes coated with Ag/GrO nanocomposites seem to be able to reduce from 106 colony forming units (CFUs) of either *E. coli* or *S. aureus* per mL to zero (Bao et al., 2011).

On the other hand, to our notice, AuNPs have never been used for the development of NP-based contraptions, and we think that such phenomenon may be explained by the reasoning that these NPs do not possess strong antimicrobial activities on their own, rather functioning as drug carriers and deliverers. As such, designing devices based upon AuNPs may be more complicated and prone to dysfunctions, but research is needed to discard its potential uses in such field. An interesting potential application for this kind of NPs is its conjugation with existing disinfectants, as it may potentiate their absorption and ultimately enhance their efficiency. Moreover, silver and/or graphene NP-based disinfectants could act as standalone disinfectants, as they have a proven antimicrobial activity, and as disinfectants are used on inert surfaces the requirements for safety are lower than those for drugs.

On the other hand, even when the green synthesis methods for NP production using plants (Ovais et al., 2018b) and microbial enzymes (Ovais et al., 2018a) have been considered highly cost-effective and eco-friendly, NPs that are produced by top down, and especially, bottom up techniques have an edge on size consistency, sizing accuracy, and shaping (Slepička et al., 2019). Thus, if a especial shape and/or size is needed in order for the NP-modified device to work properly, cost may be a limitation for the technology. In this sense, research on the improvement of the so called green-synthesis methods may be a key to empower NP-coated devices.

## Conclusion

Taken together, the aforementioned data suggest that NPs may be effective allies for fighting AR-bacteria, as they are not only effective against several regular bacterial species, but they have also shown good antimicrobial abilities against AR-strains. Their properties to fight AR-bacteria even extend to the ability of graphene NPs to degrade r-plasmids and to inhibit the expression of r-genes, which may limit the spread and development of AR. Nonetheless, such substances have been shown to possess a certain degree of toxicity, which renders them as poor candidates for drug development. For this reason, NPs may be best suited for the development of extracorporeal devices with antimicrobial properties, as this kind of contraptions may utilize their potential for fighting AR-pathogens without compromising human health.

Much research is needed in order to fully evaluate the potential use of the aforementioned devices in the clinical setting, but most of the research in this area aim at the development of biomedical contraptions, without extrapolations into other endemic zones for AR-bacteria. Thus, we think that veterinary hospitals, farms and sewages must not be neglected in these initiatives, as they represent important sources of AR-bacteria, and a complete environmental control of AR-pathogens in such endemic zones may be of use in the fight against the phenomenon.

Much research is needed regarding this topic as the durability of the antimicrobial coatings, the proper NP density for their optimal function, whether or not they produce nano-contamination, or even the effectiveness and safety of other NP-based contraptions (like NP-based disinfectants) have not been studied. Also, further studies warrant a correct extrapolation of the resulting devices in all the AR-endemic zones in order to provide further control of the AR-bacteria.

## Author Contributions

MG-N: NPs physical, chemical, and biological properties and NP-based devices. MC-L: NPs physical, chemical, and biological properties. FS-C: NP-based devices. OR-R: NP-toxicity. FR-L: Introduction and epidemiology. DM-S: epidemiology. RM-D: resistance to NPs. AP: study design and original idea and supervision of the writing. All authors contributed to the article and approved the submitted version.

## Conflict of Interest

The authors declare that the research was conducted in the absence of any commercial or financial relationships that could be construed as a potential conflict of interest.
